# Host-specific probiotics feeding influence growth, gut microbiota, and fecal biomarkers in buffalo calves

**DOI:** 10.1186/s13568-022-01460-4

**Published:** 2022-09-14

**Authors:** Vinay Venkatesh Varada, Sachin Kumar, Supriya Chhotaray, Amrish Kumar Tyagi

**Affiliations:** 1 Rumen Biotechnology Lab, Animal Nutrition Division, ICAR-National Dairy Research Institute, Karnal, 132001 Haryana India; 2 Buffalo Breeding Lab, Animal Genetics and Breeding Division, ICAR-National Dairy Research Institute, Karnal, 132001 Haryana India; 3 Present Address: Assistant Director General (Animal Nutrition and Physiology), Indian Council of Agricultural Research, New Delhi, India

**Keywords:** Early-life, *Lactobacillus *spp., Metagenomics, Gut microbiota, Murrah calves

## Abstract

The current study is aimed to evaluate the effect of host-specific probiotics on the gut microbiome, performance, and select fecal biomarkers of gut health in preruminant buffalo calves. Eight Murrah buffalo calves (3–5 days old; 32.52 ± 0.43 kg average body weight (BW)) were randomly allocated into two groups as follows; 1) Group I (n = 4) fed basal diet alone (CON); 2) Group II (n = 4) supplemented with a lyophilized probiotic formulation at a dose rate of 1 g/day/head (1 × 10^9^ CFU/g) having *Limosilactobacillus reuteri* BF-E7 and *Ligilactobacillus salivarius* BF-17 along with basal diet (PF) for 30 days. Results revealed that final BW (kg), average daily gain (g/day), average dry matter intake (g/day), and structural growth measurements were significantly (P < 0.05) increased in the probiotics supplemented group (PF) compared to the control (CON). Fecal pH, fecal moisture, and fecal score were reduced (P < 0.05) in PF than in CON. Moreover, levels of fecal propionate, lactate, and ammonia altered positively in PF compared with CON. The relative abundance of *Firmicutes* tended to be higher (P = 0.10) in the probiotics fed group than CON. However, the relative abundance of *Proteobacteria* was significantly lower (P = 0.03) for calves fed probiotics on day 15. A trend was observed in *Bacteroides* (P = 0.07) and *Lactobacillus* (P = 0.08) abundances in the feces of the PF than in CON. Overall, it can be concluded that the administration of probiotic formulations significantly improved the performance and gut health of buffalo calves via modulating the gut microbiota composition.

**Graphical Abstract:**

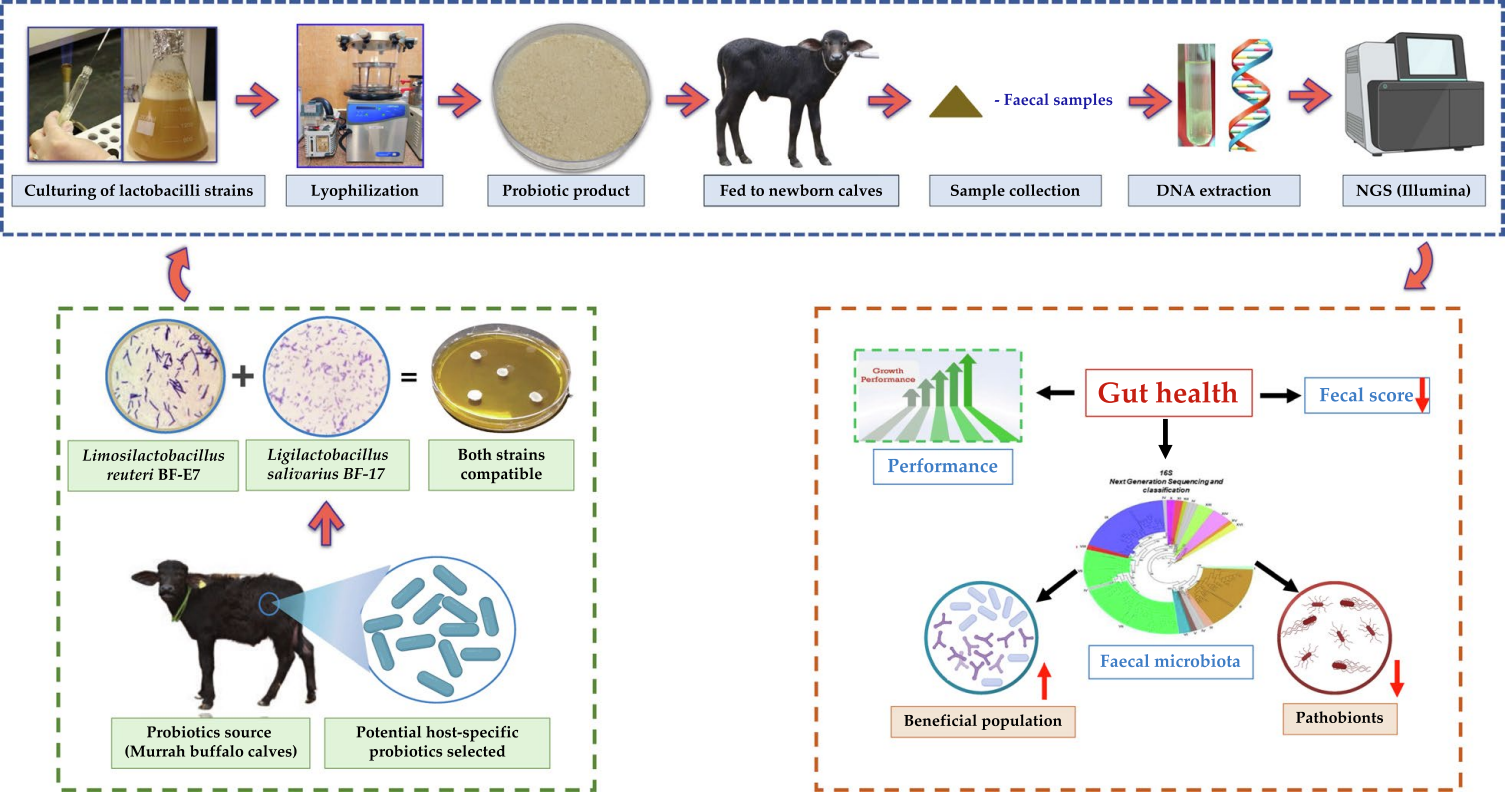

**Supplementary Information:**

The online version contains supplementary material available at 10.1186/s13568-022-01460-4.

## Introduction

The gastrointestinal tract (GIT) of the calf undergoes a dramatic morphological and metabolic transformation in the initial few weeks of life (Arshad et al. 2021). The transition of calves from a pseudo-monogastric phase (non-functional rumen or preruminant) to an active rumination phase is recognized as the most challenging physiological stage (Amin et al. 2021). The establishment of health-promoting bacteria in the GIT during early life promotes efficient feed digestion, nutrient absorption, and energy metabolism, thereby ensuring proper intestinal maturation (Celi et al. 2017). However, swift shifts in diet (from a highly palatable whole milk-based diet to a poorly digestible plant-based solid food), an altered environment, and social conditions in the developmental stage may lead to gut dysbiosis (Singh et al. 2021a). In particular, pre-ruminant calves are more susceptible to perturbation of enteric microbiota, which may severely affect their growth efficiency and overall productive performance in the later part of life (Fernández et al. 2020). Consequently, gastrointestinal disorders like neonatal calf diarrhea (NCD) may occur during the first four weeks, resulting in high morbidity and mortality rates (up to 20% of total calf death), causing tremendous economic and productivity losses in dairy farms worldwide (Sreedhar et al. 2010; Masucci et al. 2011). Conventional antimicrobials are given in prophylactic (for growth-promoting effect) and therapeutic doses to curb calf scour and promote livestock production (Jiang et al. 2020). However, the untargeted use of antibiotics has become a global health threat due to the emergence of antimicrobial resistance and may negatively impact the gut microbial composition of the calf. Hence, there is a renewed interest among the global scientific fraternity in developing natural, safe, effective, and sustainable substitutes for antimicrobials to improve bovine health and welfare. In this case, probiotics appear to keep the intestinal environment stable, boost the immune system, and reduce diarrheal episodes in calves during their first years of life (Renaud et al. 2019).

Accumulating research evidence has revealed the potential role of early-life gut microbiota in shaping and programming the host mucosal immune system and its long-term impacts on metabolic consequences in adult life (Choudhury et al. 2021; Rosa et al. 2021; Wu et al. 2021). This intricate knowledge of the host-microbial relationship may provide a ‘window of opportunity’ for the successful manipulation of the gut microflora using dietary interventions. In light of this, any optimal nutritional strategies targeted to support beneficial gut microbiota proliferation could aid in enhancing animal health and productivity (Arshad et al. 2021). Timmerman et al. (2004) emphasized that multispecies probiotics exert a myriad of nutritional and health benefits and are more effective than single-species probiotics. However, the efficacy of probiotics is dependent on the niche of origin, the microbial strain selected, and their combination, considering their effects are likely to be host-strain-specific (Singh et al. 2021b; Sanders et al. 2018). An increasing number of studies have reported that multispecies probiotic supplementation confers numerous health benefits, including improved growth performance and gut health indices of animals, thus indicating its remarkable potential to be used as a microbial feed additive in calf nutrition (Cangiano et al. 2020; Wu et al. 2021; Guo et al. 2022). Various researchers have also successfully demonstrated the efficacy of host-specific (autochthonous) probiotics in poultry (Reuben et al. 2022), calves (Fernández et al. 2020; Varada et al. 2022), canines (Kumar et al. 2017), and pigs (Oh et al. 2021). Given the fact that probiotics derived from the original target host have been evidently revealed to be more efficacious than other source origins (allochthonous) (Fernández et al. 2018; Kumar et al. 2022), future strategies should focus on exploiting native multispecies probiotics to reduce the severity and incidence of NCD in dairy calves.

Rapid scientific advances in the field of microbiology, combined with bioinformatic tools, have made it possible to better understand the role of diverse unculturable microbes in complex econiche (Choudhury et al. 2021). For instance, metagenomics has enhanced our knowledge about intestinal microbial communities and aided in identifying, characterizing, and developing host-specific probiotics from the calf gut (Zhang et al. 2021). There is a need to elucidate the underlying core mechanisms by which probiotics influence gut microbial communities in young ruminants. Nevertheless, the beneficial response of probiotic supplementation on the modulation of gut microbes in newborn calves is inconsistent and their functional activities remain unexplored. This, in turn, highlights the need for further in-depth research to unravel the impact of early-life GIT microbial colonization and its role in shaping intestinal physiology, which is of great significance for dairy calf health. To the best of our knowledge, studies on the effects of host-specific probiotics on gut microbial communities using high-throughput sequencing technologies in buffalo calves are not reported earlier. However, several studies have shown the beneficial effects of *Lactobacillus* spp. on gut microbiota development and to minimize calves’ susceptibility to enteric infections during the pre-weaning period (during the first 4 weeks of life) (Fomenky et al. 2018; Wu et al. 2021; Guo et al. 2022). These results also include improved body weight and a reduction in the occurrence of diarrhea (Lu et al. 2022). Based on this, we hypothesized that the administration of host-specific *Limosilactobacillus reuteri* BF-E7 and *Ligilactobacillus salivarius* BF-17 might improve the performance and gut health of preruminant calves. Therefore, the present study was conducted to investigate the effects of two host-specific probiotic-based formulations on the composition and diversity of the gut microflora and performance in Murrah buffalo calves.

## Materials and method

### Probiotic strains and lyophilization

The two potential probiotic strains (*Limosilactobacillus reuteri* BF-E7, GenBank Accession No-MG966332 and *Ligilactobacillus salivarius* BF-17, GenBank Accession No-MG966326) used in this study were isolated from healthy newborn Murrah buffalo calves’ feces in the previous experiment (Singh et al. 2021b). In vitro analysis showed promising techno-functional probiotic attributes for the two lactobacilli strains. The selected strains were subcultured and reactivated in de Mann Rogosa and Sharpe broth (MRS; HiMedia Laboratories, Mumbai, India) at 37 °C for 24 h. The cross-streak method and culture-spot technique revealed that both the probiotic strains were compatible in vitro as there was no inhibition zone observed against each other on the MRS agar plates (Additional file 1: Figure S1). Both the activated *Lactobacillus* cultures were inoculated into MRS broth (1 L) at 2% (v/v) and incubated under the above-mentioned conditions. The bacterial cell pellets were harvested after centrifugation (10000*g* for 10 min at 4 °C), then washed twice with 50 mL of sterile phosphate buffer saline and lyophilized according to Fernández et al. (2018) and Varada et al. (2022) with minor modifications. The standard plate count method was used to enumerate the bacterial numbers [colony forming unit (CFU/g)] of the lyophilized probiotic product (Bayatkouhsar et al. 2013). The prepared probiotic product was used for the experimental feeding trial.

### Animals and feeding management

**Fig. 1:**
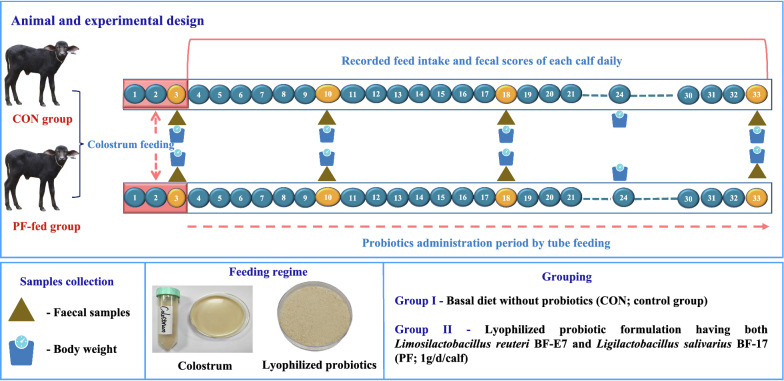
Animal, experimental design, feeding regime, and sample collection of the current study in Murrah buffalo calves supplemented with probiotic product. Calves were fed colostrum up to 3 days after birth. From day 4 to 33 of age, calves were administered with probiotics by tube feeding. Body weight was recorded at day 0, 7, 14, 21, and 30 of experimental days. At day 3, 10, 18, and 33 of age, fecal samples were collected and bacterial composition of each calf was measured. From day 4 to 33 of age, fecal scores were recorded daily for each calf

Eight healthy neonatal Murrah buffalo calves (3–5 days old; 32.52 ± 0.43 kg average BW) were randomly allocated to two treatment groups after completing the colostrum feeding period based on body weight as follows; 1) Group I (n = 4) fed basal diet alone (CON); 2) Group II (n = 4) supplemented with a lyophilized probiotic formulation at a dose rate of 1 g/day/head (1 × 10^9^ CFU/g) having *Limosilactobacillus reuteri* BF-E7 and *Ligilactobacillus salivarius* BF-17 along with basal diet (PF) for 30 days. The calves were housed in individual pens (1.6 × 2.5 m) bedded with wood shavings at the Livestock Research Centre farm (LRC, National Dairy Research Institute, India). The calf pens were cleaned, renewed twice a day (removing manure) and disinfected using diluted phenyl solution thrice weekly. Prior to the start of the four-week trial experiment, each animal was checked for any signs of disease, dehydration, or injury, and those that were initially considered unhealthy were not included as part of this study. The lyophilized probiotic formulation product containing at least 1 × 10^9^ CFU/g was dissolved into sterilized skim milk powder (20% w/v), reconstituted in distilled water and fed once a day to the PF group in the morning by tube feeding for 30 days. All the buffalo calves had ad libitum access to a calf starter diet (from 7 days onwards), green forage (maize and sorghum), and fresh clean water throughout the experimental period according to the feeding regimen of Sharma et al. (2018). The calf starter (concentrate mixture) was formulated using quality ingredients, and the composition is given in Table 1. The design of this experiment is shown in Fig. 1.

**Table 1:** Chemical composition (on % DM basis) of the basal diet^†^ and milk fed to Murrah buffalo calves

Nutrients	Calf starter (concentrate mixture)	Green fodder	
		Maize	Sorghum
DM	90.16	26.18	25.18
OM	92.80	89.62	92.59
CP	23.33	9.43	8.32
EE	4.59	2.71	1.56
NDFom	26.89	65.36	63.81
ADFom	16.73	31.55	33.38

Premix provided per kilogram of concentrate: vitamin A, 15,000 IU; vitamin D, 5000 IU; vitamin E, 50 mg; Fe, 90 mg; Cu, 12.5 mg; Mn, 30 mg; Zn, 90 mg; Se, 0.3 mg; I, 1.0 mg

*DM* dry matter, *OM* organic matter, *CP* crude matter, *EE* ether extract, *NDFom* neutral detergent fibre corrected for ash, *ADFom* acid detergent fibre corrected for ash

*Milk composition: Whole milk (16.48% DM, 7.84% fat, 9.4% SNF), Skim milk powder (94.3% DM, 1.19% fat)

^†^Ingredients proportions (%): 29 maize, 16 soybean meal, 14 wheat bran, 14 mustard oil cake, 10 rice polish, 9 ground nut cake, 5 bajra, 2 vitamin and mineral premix, 1 salt

### Growth performance and structural growth measurements

The BW of the calves was recorded weekly using an automated electronic weighing scale before morning feeding. The feed offered and orts plus wastage were measured daily to determine the dry matter intake (DMI) of individual calves. Weekly measurements of structural growth parameters, viz*.,* hip height, body length, heart girth, and wither height, were recorded by employing Lesmeister et al. (2004) techniques. Average daily gain (ADG) and feed efficiency (FE) were calculated later. Proximate principles of feed were analyzed as per the standard procedures of the Association of Official Analytical Chemists (AOAC, 1995).

### Feces collection, sampling, and select gut health biomarkers estimation

Fresh fecal samples of each animal were scored twice daily for consistency and appearance by employing a five-point fecal scoring system, where 1 = firm feces, 2 = normal or firm-soft feces, 3 = moderate or soft feces, 4 = mild or runny diarrhea, and 5 = watery and profuse diarrhea, as described by Magalhaes et al. (2008) with minor modifications. Fecal samples from individual calves were collected in duplicate into autoclaved centrifuge tubes at day 0, day 15, and day 30 of the experiment using sterile gloves after manual stimulation by rectal ampulla. A digital pH meter was used to measure fecal samples’ pH, specially designed for direct pH measurement of semi-solid samples (pH Spear; Eutech Instruments, Klang Selangor, DE, Malaysia; pH Range: −1.00 to 14.00 pH, Resolution: 0.01 pH, Accuracy: ± 0.01 pH). For estimation of fermentative end products, namely lactate, ammonia, and short-chain fatty acids (SCFA), standard protocols were adopted as described in an earlier publication (Kore et al. 2009). Briefly, about 6 mL of 6.0 N HCl was added to 2.0 g of fresh faeces and preserved at –20 °C to analyse ammonia later. A volume of 4 mL of metaphosphoric acid (25% w/v) was mixed with 2 g of fresh feces, followed by centrifugation (10000*g*) for 10 min. The resulting supernatant was stored at –20 °C for analysis of SCFA. For lactate analysis, the third aliquot of about 2 g of fresh feces was diluted with 4 mL of distilled water and centrifuged at 10,000 g, and the supernatant was stored at −20 °C until further processing.

### Fecal DNA extraction and purification

Fresh fecal samples were collected on different days (day 0, day 7, day 15, and day 30) aseptically to investigate intestinal bacterial composition using sterile gloves and lubricant from individual calves into sterile cryogenic vials (DNase-RNase free; Corning^®^, USA). According to the manufacturer's protocol, bacterial genomic DNA was extracted from feces (0.2 g) using the commercial kit (QIAamp^®^ Fast DNA Stool Mini Kit, QIAGEN Inc., Valencia, CA, USA). After extraction, we purified the genomic DNA using the QIAamp 96 PowerFecal QIAcube HT Kit (QIAGEN Inc., Valencia, CA, USA) to obtain higher yields, better purity, and a more accurate representation of the microbial diversity. DNA purity and quantity were checked on agarose gel electrophoresis (2.0%) and using a NanoDrop 1000 spectrophotometer (Thermo Scientific, Wilmington, DE, USA), and the DNA samples were ready for high-throughput sequencing.

### Microbial profiling using Illumina miseq sequencing

In this experiment, the extracted DNA samples (in triplicates) were subjected to PCR amplification targeting the highly variable V3-V4 region of the bacterial 16S rRNA gene (forward primer, 338F, 5′-ACTCCTACGGGAGG CAGCA-3′; reverse primer, 806R, 5′-GGACTACHVGGGTWTCTAAT-3′). The detailed amplification and sequencing protocol were consistent with the previously published report (Liu et al. 2019). The sequencing was carried out on an Illumina MiSeq PE250 platform. Raw 16S rRNA gene amplicon sequence FASTQ files were checked for quality, and sequences with low-quality bases and adapter sequences were removed. Initial Operational Taxonomic Unit (OTU) picking and taxonomic assignment from representative sequences of OTUs were performed using QIIME v1.8.0. Sequences from all the samples were picked using close reference and clustered into OTUs at 97% sequence similarity with UCLUST. Rarefication was performed to normalize library size for the amplicon sequence data to remove the samples with small library sizes (Weiss et al. 2017). A rarefaction curve for each sample was plotted (observed OTUs metric) in order to choose the rarefaction threshold for all samples and to ensure that sampling depth was sufficient for the analysis of each sample (Additional file 1: Figure S2). Results were generated after total sum scaling normalization method i.e. percentage abundance = (counts for each taxa *100)/the total counts for the sample prior to statistical analysis. Relative counts of different OTUs were determined for downstream statistical analysis. The R package “*phyloseq*” was used to construct a physeq object based on the absolute count and taxonomic groups identified. A stacked bar plot was created at both phylum and genus levels to visualize the relative abundance on various days of probiotic treatment versus control at different time points. The R package “*microbiome*” was used to determine bacterial community composition and to generate various diversity indices (Shannon and Simpson) and richness indices (Chao 1) of the fecal microbiota. Core genera were identified using the R package “microbiome” with a detection rate of 0.001 in at least 90% of the samples and a 0.75 prevalence. Core genera in the control and treatment groups were identified separately at different days of experimentation and were represented through Venn diagrams (Zaura et al. 2009). Similarly, the shared core members at different days of experimentation for control and treatment groups were presented separately. Differences between samples in the groups and at different days in the microbial community were assessed using the Bray–Curtis dissimilarity at the genus level using the “*phyloseq*” package and the results were presented in the non-metric multi-dimensional scaling (NMDS) plot.

### Statistical analysis

The SPSS software (Statistical Package for the Social Sciences, Version 21.0, Chicago, IL, USA) was used to analyse the experimental data. The data of parameters collected periodically were analyzed using the analysis of variance (ANOVA) method to determine statistical significance. The values were presented as means with a standard error of the mean (SEM). All results were considered statistically significant if P < 0.05 and 0.05 ≤ P < 0.10 were regarded as a statistical tendency.

## Results

### Growth performance and structural body measurements

At the beginning of the study, no significant difference was observed in the initial BW between the two groups. However, the lyophilized probiotic formulation fed group’s final BW (kg) (P = 0.01), net gain (kg) (P = 0.028), ADG (g/d) (P = 0.029), and average DMI (g/d) (P = 0.001) were significantly higher than the control group (Table 2). The feed efficiency tended to be higher (P = 0.096) in the PF group compared to the control group. The PF-fed calves had considerably increased body length (P < 0.001), heart girth (P = 0.003), wither height (P = 0.001), and hip height (P < 0.001) compared with the control group (Table 2).

**Table 2:** Growth performance and structural measurements of Murrah buffalo calves supplemented with probiotic formulation (PF) or not (CON)

Item	Treatment (Trt)^1^		SEM	P Value (Trt)
	CON	PF		
Initial BW (kg), d0	32.55	32.49	0.43	0.953
Final BW (kg), d30	37.71^a^	40.15^b^	0.56	0.011
Net gain in BW (kg)	5.16^a^	7.66^b^	0.62	0.028
^2^ADG (kg/d), 0–30 d	0.18^a^	0.26^b^	0.02	0.029
^3^ADMI (kg/d), 0–30 d	0.46^a^	0.51^b^	0.01	0.001
^4^Feed efficiency %	37.44	50.30	4.12	0.096
^A^Structural growth measurements				
Body length (cm)	55.97^a^	58.82^b^	1.41	< 0.001
Heart girth (cm)	80.68^a^	82.23^b^	1.23	0.003
Wither height (cm)	72.76^a^	74.04^b^	0.98	0.001
Hip height (cm)	74.47^a^	76.44^b^	1.02	< 0.001

*BW* body weight, *ADG* average daily gain, *ADMI* average dry matter intake, *Trt* treatment, *SEM* standard error of mean

^1^Treatments: ^†^Basal diet with no supplementation (CON), supplemented with *L. reuteri* BF-E7 + *L. salivarius* BF-17 (PF; 1 g/calf/day)

^ab^Means bearing different letters in a row differ significantly (*P* < 0.05)

^A^Mean of four periodic collections; P < 0.05 was regarded as statistically significant; ^2^ADG = (kg of final BW—kg of initial BW)/experimental days

^3^ADMI = (offered DM—residual DM)/experimental days

^4^Feed efficiency = [Average daily gain (kg/day)/Dry matter intake (kg/day)] × 100%

### Gut health biomarkers

**Fig. 2:**
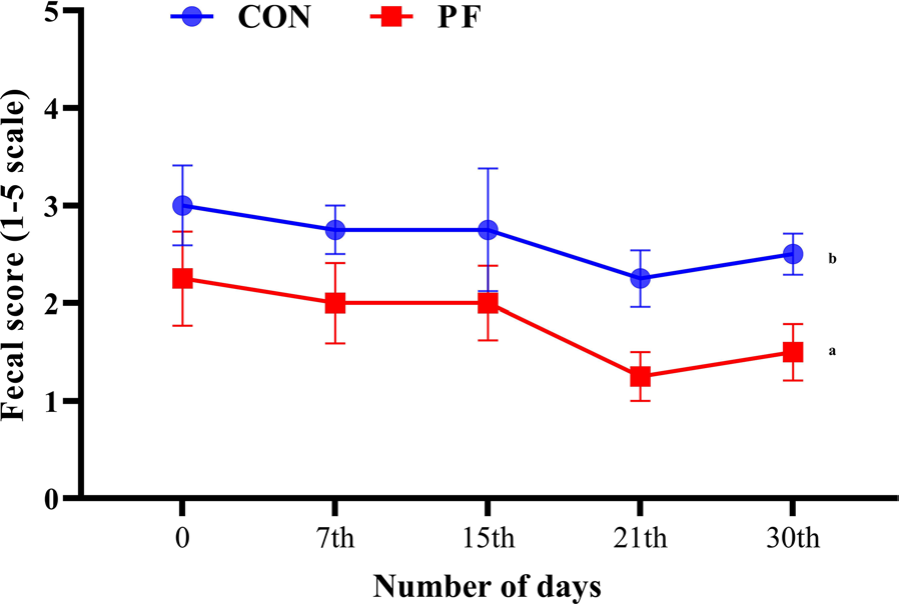
Fecal score of Murrah buffalo calves supplemented with probiotics as compared to control group (significance: T = 0.001; D = 0.193; T × D = 0.992). Basal diet with no supplementation (CON), supplemented with *L. reuteri* BF-E7 + *L. salivarius* BF-17 (PF; 1 g/calf/d). Fecal score was noted using 1–5-point scale (1 = firm faeces, 2 = normal or firm-soft faeces, 3 = moderate or soft faeces, 4 = mild or runny diarrhea, and 5 = watery and profuse diarrhea)

The effects of PF supplementation on the gut health biomarkers in neonatal buffalo calves are shown in Table 3. Notably, the average fecal pH (P = 0.004) and fecal moisture (P < 0.001) were remarkably lower in the PF-fed group in comparison to the control group. Fecal scores of the PF group were significantly lower (P = 0.001) when compared with the control group with the progressing age of calves (Fig. 2). Evaluation of fecal metabolites indicated a statistical difference (P < 0.001) in lactate concentration between the PF and CON groups. In contrast, ammonia levels in PF-fed calves were significantly lower (P = 0.001) than in the control group. However, no differences were found (P > 0.05) in mean fecal acetate and butyrate concentrations between PF-fed calves and control group, whereas the propionate concentration was significantly higher (P = 0.026) in the former than in the latter.

**Table 3:** Fecal characteristics, metabolites, and short chain fatty acids of Murrah buffalo calves supplemented with probiotic formulation (PF) or not (CON)

Item	Treatment (Trt)^1^		SEM	P Value (Trt)
	CON	PF		
^A^Fecal characteristics				
Fecal pH	7.44^b^	7.10^a^	0.23	0.004
^ 2^Fecal score	2.75^b^	1.92^a^	0.45	0.030
Fecal moisture (%)	84.56^b^	82.42^a^	1.49	< 0.001
^A^Fecal metabolites (µmol/g of fresh feces)				
Ammonia	6.01^b^	5.30^a^	0.39	0.001
Lactate	3.32^a^	4.07^b^	0.30	< 0.001
^B^Fecal short chain fatty acids (µmol/g of fresh feces)				
Acetate	28.56	28.74	1.12	0.808
Propionate	10.75^a^	12.08^b^	0.75	0.026
Butyrate	4.60	4.82	0.45	0.433

*Trt* treatment, *SEM* standard error of mean

^1^Treatments: ^†^Basal diet with no supplementation (CON), supplemented with *L. reuteri* BF-E7 + *L. salivarius* BF-17 (PF; 1 g/calf/day)

^ab^Means bearing different letters in a row differ significantly (*P* < 0.05)

^A^Mean of three periodic collections

^B^Mean of two periodic collections; P < 0.05 was regarded as statistically significant

^2^Fecal score rating: 1 = firm feces, 2 = normal or firm-soft, 3 = moderate or soft, 4 = mild or runny diarrhea, and 5 = watery and profuse diarrhea

### Diversity and taxonomic composition of microbiota in the feces

**Fig. 3:**
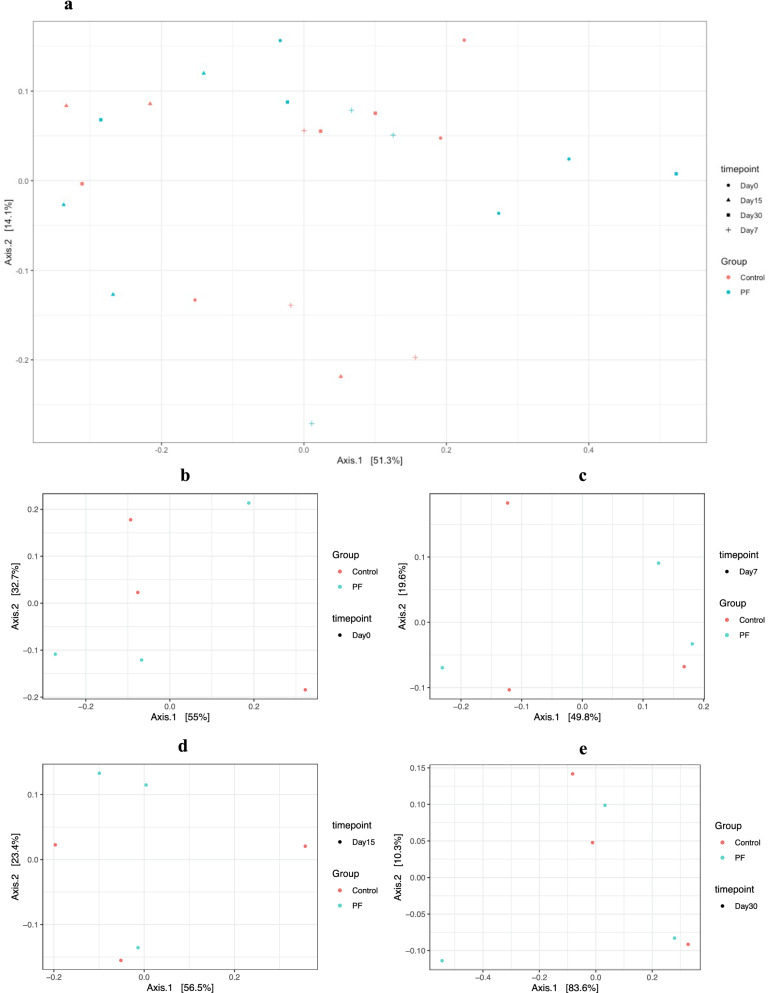
Change in the fecal bacterial community structure in Murrah buffalo calves from 0 to 30 days of age. The non-metric multi-dimensional scaling (NMDS) plot was generated based on Bray–Curtis dissimilarities of fecal bacterial community determined via 16S rRNA gene amplicon sequencing (**a** Overall 30 days, **b** Day 0, **c** Day 7, **d** Day 15, **e** Day 30). Colours indicate the dietary groups; control (red) and PF (blue). Different symbols represent different age points. Individual points represent individual animals. Basal diet with no supplementation (CON), supplemented with *L. reuteri* BF-E7 + *L. salivarius* BF-17 (PF; 1 g/calf/d)

**Fig. 4:**
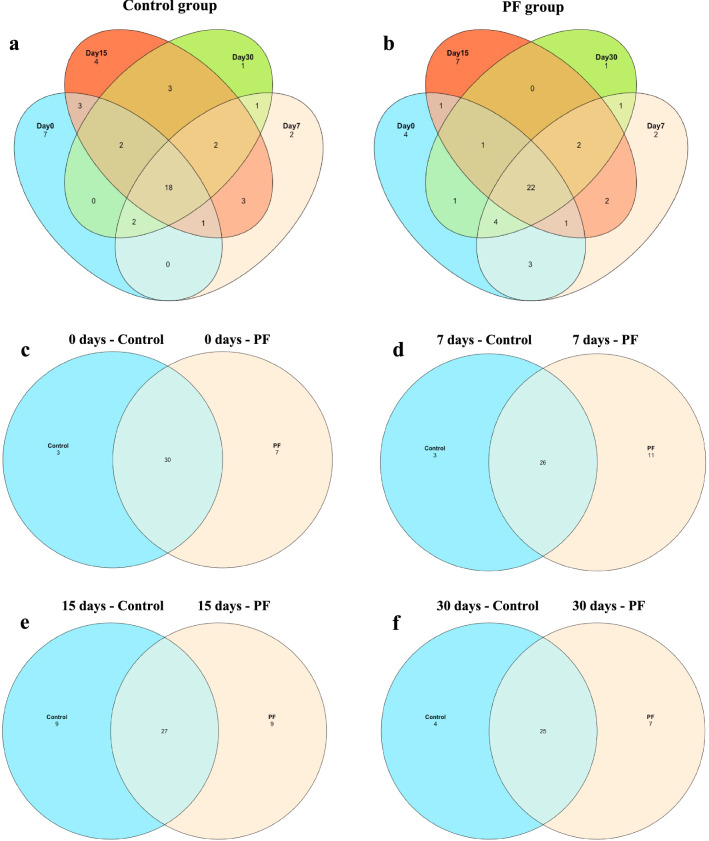
The Venn diagrams show the number of core operational taxonomic units that were shared or not shared by the control group and treatment group individuals, depending on overlap. The common and specific genera in the **a** Control group. **b** PF group. **c** Day 0. **d** Day 7. **e** Day 15. **f** Day 30. Core genera were identified with a detection threshold of 0.001 in at least 90% of the samples and a 75% prevalence. Basal diet with no supplementation (CON), supplemented with *L. reuteri* BF-E7 + *L. salivarius* BF-17 (PF; 1 g/calf/d)

The results of alpha-diversity showed that no significant differences (P > 0.05) in Shannon, Simpson, and Chao1 indices were found between the PF-fed and control groups (Additional file 1: Figure S3). The NMDS plot shows that the axis 1 contributes maximum to the Eigenvalues thus representing the diversity between samples. At the extreme point of the axis 1, the sample of 30th day PF group lies indicating its distance from other samples in terms of the composition of various genera (Fig. 3). A total of 123 taxa were analysed to identify the core fecal microbiomes of the animals. The components shared by all individuals in each sample or group were considered to be the core bacterial communities. The core fecal microbiota between the control and PF groups at different time points (Day 0, 7, 15, and 30) and was presented in the form of a Venn diagram (Fig. 4). The PF-fed calves shared a larger number of unique bacterial OTUs than those in the control group at 7 and 30 days of experiment (PF vs. CON, 11 vs. 3 and 7 vs. 4, respectively).

**Fig. 5:**
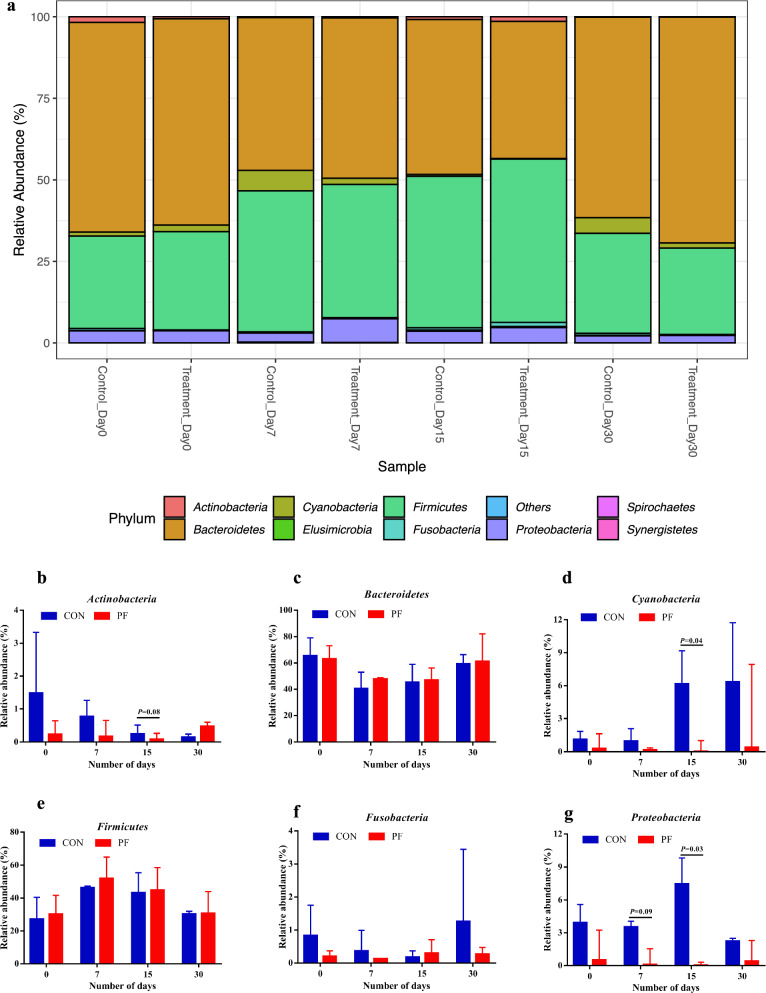
Effects of PF on the relative abundance of faecal bacterial composition of Murrah buffalo calves at the phylum level. **a** Phylum level composition. **b** The change of *Actinobacteria.* **c** The change of *Bacteroidetes.* **d** The change of *Cyanobacteria.* **e** The change of *Firmicutes.* **f** The change of *Fusobacteria.* **g** The change of *Proteobacteria.* Color-coded bar plot showing the relative abundances across different treatments at four time points during the study (Day 0, 7, 15, and 30). Each bar represents the top ten bacterial phyla ranked by the relative abundance in each individual sample or group. P values < 0.05 were considered significant, P values between 0.05 and 0.10 were considered as a tendency. Values are expressed as mean ± SD. Basal diet with no supplementation (CON), supplemented with *L. reuteri* BF-E7 + *L. salivarius* BF-17 (PF; 1 g/calf/d)

**Fig. 6:**
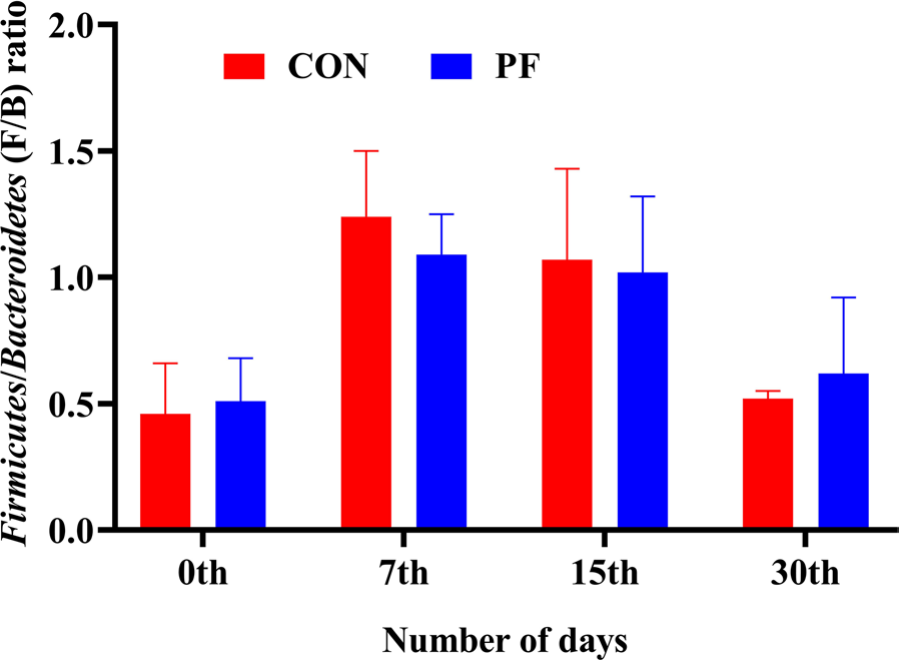
The ratio of *Firmicutes*/*Bacteroidetes* (F/B) in the feces of Murrah buffalo calves supplemented with probiotics as compared to control group. Basal diet with no supplementation (CON), supplemented with *L. reuteri* BF-E7 + *L. salivarius* BF-17 (PF; 1 g/calf/d)

The taxonomic profiling of 16S amplicon sequencing data revealed a total of six major phyla were identified in both the groups from 24 fecal samples (12 from CON and 12 from PF) of buffalo calves (Fig. 5a and Additional file 1: Table S1). Among them, the *Bacteroidetes* and *Firmicutes* were the most predominant phyla in the feces of neonatal calves, and their abundance ranged from 26.69% to 75.23% and 18.55% to 65.11%, representing an average of > 50% of the total population. *Bacteroidetes* accounted for more than 54% and 52% of total bacteria in the PF and control groups, respectively (Fig. 5c). The relative abundance of *Cyanobacteria* (Fig. 5d) was significantly lower (P = 0.04) on day 15 in the PF-fed group (1.61%) than in the control group (6.15%). The average relative abundance of *Firmicutes* (Fig. 5e) tended to be higher (P = 0.10) in the PF-fed group than in the control group (PF vs. CON, 39.44% vs 33.29%). On day 15, a lower (P = 0.03) relative abundance of *Proteobacteria* was noted in the PF-fed (3.48%) group than in the control (4.28%) (Fig. 5g). However, as the treatments continued and the calves grew older, no significant changes (P > 0.05) were observed in the relative abundance across the other prevailing phyla (*Actinobacteria* and *Fusobacteria*). No significant changes were observed between the PF and control groups with respect to the ratio of *Firmicutes*/*Bacteroidetes* (F/B) ratio at different time points (Fig. 6).

**Fig. 7:**
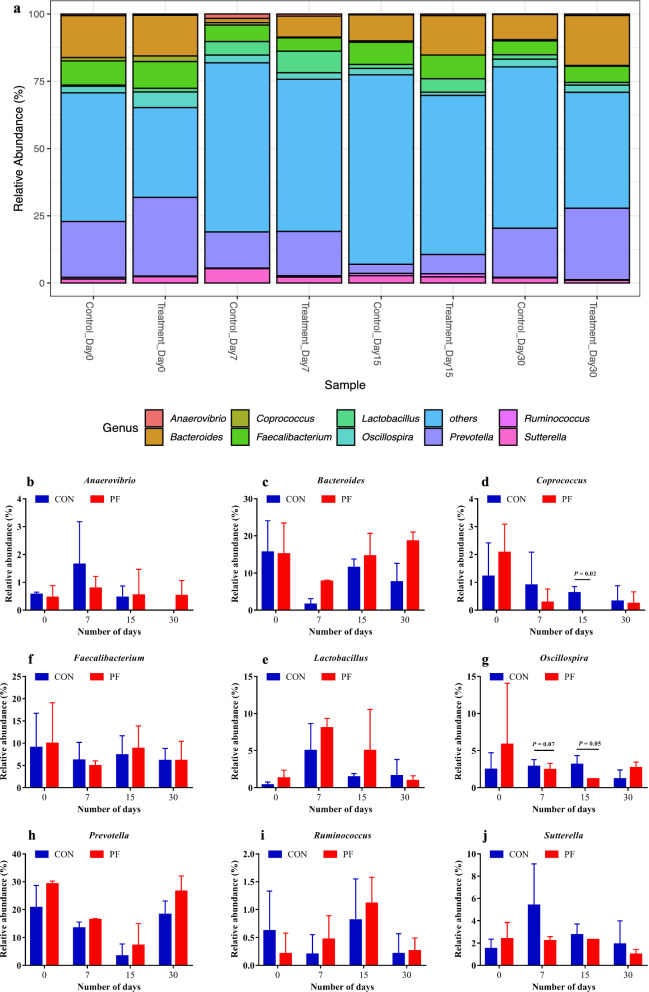
Effects of PF on the relative abundance of faecal bacterial composition of Murrah buffalo calves at the genus level. **a** Genus level composition. **b** The change of *Anaerovibrio.* **c** The change of *Bacteroides.* **d** The change of *Coprococcus.* **e** The change of *Faecalibacterium.* **f** The change of *Lactobacillus.* **g** The change of *Oscillospira.* **h** The change of *Prevotella.* **i** The change of *Ruminococcus.* **j** The change of *Sutterella.* Color-coded bar plot showing the relative abundances across different treatments at four time points during the study (Day 0, 7, 15, and 30). Each bar represents the top ten bacterial genera ranked by the relative abundance in each individual sample or group. P values < 0.05 were considered significant, P values between 0.05 and 0.10 were considered as a tendency. Values are expressed as mean ± SD. Basal diet with no supplementation (CON), supplemented with *L. reuteri* BF-E7 + *L. salivarius* BF-17 (PF; 1 g/calf/d)

**Fig. 8:**
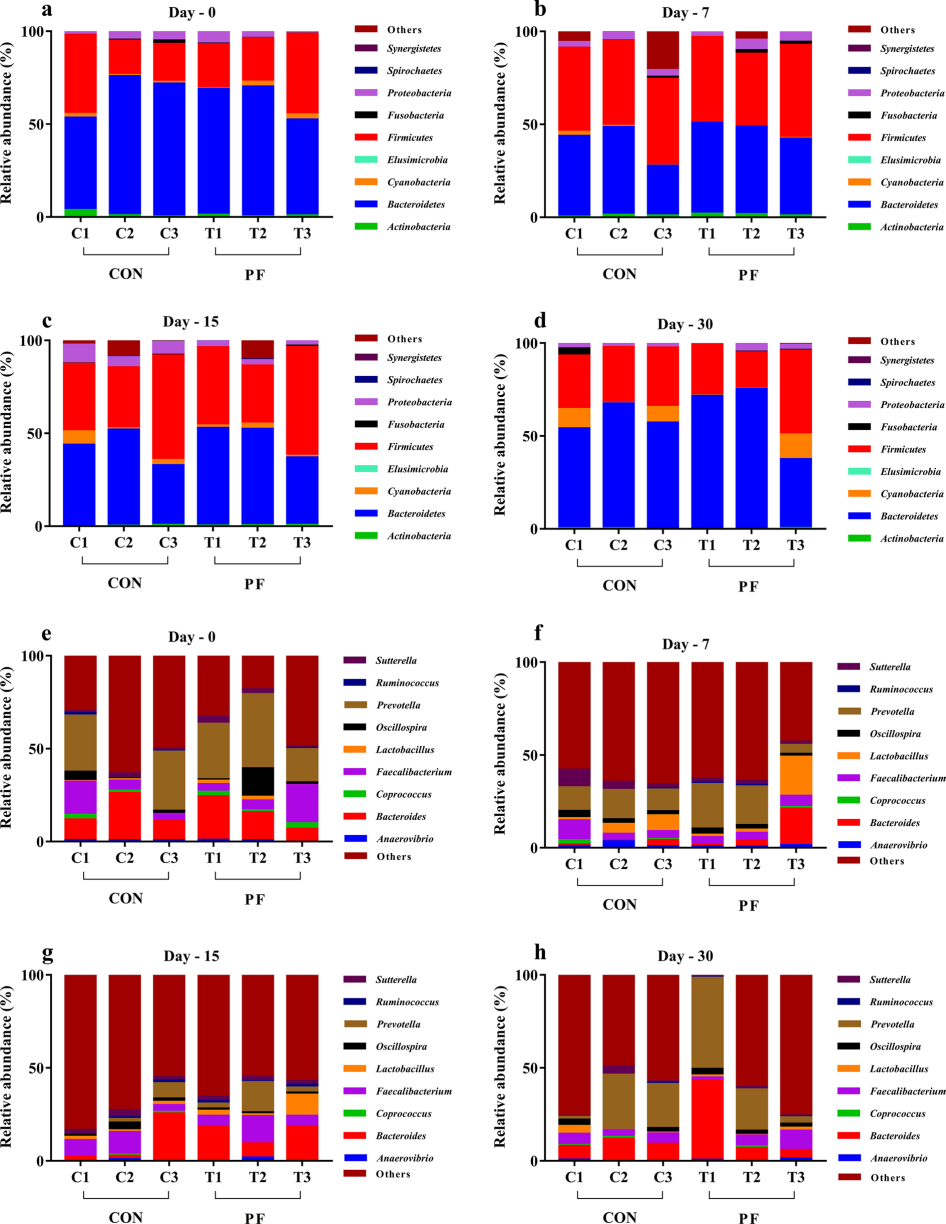
Change in the relative abundance of fecal bacterial composition of calves determined via 16S rRNA gene amplicon sequencing. Different panels show the composition of the microbial community between the two groups (CON and PF) with respect to the phyla (**a, b, c,** and **d**) and genera (**e, f, g,** and **h**) of bacterial. The animal-wise difference between the control and treatment groups at each sampling point (Day 0, 7, 15, and 30) are depicted in the graph. Basal diet with no supplementation (CON), supplemented with *L. reuteri* BF-E7 + *L. salivarius* BF-17 (PF; 1 g/calf/d)

At the genus level, a total of 16 major genera were identified in the fecal samples of buffalo calves in both groups (Fig. 7a). The average relative abundances of genus *Bacteroides* and *Lactobacillus* in the feces tended to be higher (P = 0.07 and P = 0.08) in the PF-fed group than in the control group (PF vs. CON, 14.06% vs. 9.08% and 3.88% vs. 2.1%, respectively) (Fig. 7c, e, and Additional file 1: Table S2). On day 30, the relative abundance of *Coprococcus* was considerably lower (P = 0.02) in the probiotic treated group than those in control group (Fig. 7d). The relative abundances of *Oscillospira* in PF-fed group was significantly decreased (P = 0.05) on day 15 (Fig. 7g). Furthermore, the average relative abundance of *Prevotella* was significantly higher (P = 0.01) in the PF-fed group as compared with the control group (PF vs. CON, 19.85% vs. 13.94%) (Fig. 7h and Additional file 1: Table S2). The data presented in Fig. 8 depicts the animal-wise difference between the fecal microbial community profiles of individual animals at a particular time point as direct comparison between control and treatment groups at phylum and genus level.

## Discussion

The post-natal period of ruminants is the most critical time window for appropriate development, optimal functioning, and maturation of GIT. Earlier work has recapitulated the profound impacts of early life administration of certain bacterial inocula on the performance and gut microbial communities in preruminant dairy calves (Kumar et al. 2021a; Varada et al. 2022; Wu et al. 2021). Our in vitro compatibility assay showed that *Limosilactobacillus reuteri* BF-E7 and *Ligilactobacillus salivarius* BF-17 were synergistic and complemented each other functionally, thus may exhibit additive techno-functional properties in vivo. The current study revealed that supplementation with two host-specific probiotics within four weeks of the calf’s birth resulted in a significant rise in BW gain, ADG, and morphometric measurements. We speculate that increased ADMI coupled with a trend for higher feed efficiency in the PF-fed group can promote a greater growth performance of neonatal buffalo calves. Different studies (Jiang et al. 2020; Lu et al. 2022) have shown that feeding probiotics could significantly enhance calf gut health, promote feed digestion, rumen epithelial metabolism, nutrient utilization capacity, and subsequent higher intestinal absorption of digested end products. Singh et al. (2021a) have demonstrated that an increased starter intake by calves provides a greater supply of nutrients and sufficient energy for skeletal deposition. This indicates a positive correlation between BW and biometric measurements, as Stefanska et al. (2021) reported. Consistently, other studies have declared that the inclusion of lactic acid bacteria (LAB) in the calf diet could increase ADG, body mass index, feed intake, and overall growth rate and development (Bayatkouhsar et al. 2013; Lucey et al. 2021; Wu et al. 2021), thus supporting our obtained data. Contrarily, Kumar et al. (2021a) have reported no significant differences in growth performance on feeding LAB in calves. Therefore, the multiple potential health benefits of probiotics are species-specific and rely on the composition of the bacterial inoculum (mixed strains/species), form (liquid/powder), duration, method of delivery, and dosage of probiotic administration (Timmerman et al. 2004).

Calves diarrhea is associated with a significant change in the structure of GIT microbiota and its composition (Lu et al. 2022). Despite, *Lactobacillus* spp. is known to alter the gut microbial community in human (Magne et al. 2020), piglets (Choudhury et al. 2021), and poultry (Salim et al. 2013), it is still largely unknown if host-specific lactobacilli reduce calves’ diarrhea by altering gut microbiota. Dietary supplementation of host-specific probiotics reduced the fecal scores, fecal moisture percentage, thus indicating an improved faecal matter with a firmer consistency vis‐à‐vis the control group. This result suggests the protective effects of probiotics in calves with a high risk of morbidity and mortality from diarrheal cases, thereby contributing to the economic profitability of dairy farming worldwide. It has been reported that LAB administration decreased fecal scores in young calves because probiotics prevent the colonization of diarrhea-causing pathobionts in the GIT either through the production of antibacterial compounds or by competitive displacement (Renaud et al. 2019; Villot et al. 2019). Consistent with this report, several studies found that calves fed with probiotics exhibited lower diarrheal incidences and the number of days with diarrhea (Lucey et al. 2021; Singh et al. 2021a; Kumar et al. 2021a). Prevention and control of NCD outbreaks in young calves before occurring are more cost effective, and current study have found that early intervention of probiotics has a better preventive effect (Wu et al. 2021). These effects were more pronounced during the first two months of the calf’s birth than in later stages, indicating probiotics are most efficacious in establishing gut microbiomes than stabilized ones (Malmuthuge et al. 2015; Guo et al. 2022). Therefore, in-feed supplementation of lactobacilli strains would be expected to maintain a eubiotic state in gut microbial ecology and beneficial in preventing diarrheal episodes in neonatal calves.

It is well known that LAB utilizes simple fermentable carbohydrates to produce lactic acid, and their proliferation rate determines the local pH in the intestine (Sakata et al. 2003). Singh et al. (2021a) used fecal pH as an indicator to assess gut health status in neonatal buffalo calves. Furthermore, the colonic fermentation of proteins by harmful microbes results in ammonia formation. The altered fecal metabolites (lactate and ammonia) levels might be associated with a possible shift in the pattern of fermentation (proteolysis to saccharolytic activity) and lowered urease action of gut microbes (Kumar et al. 2021b). Another possible explanation for this observation might be that PF supplementation could increase the abundance of health positive bacteria while decreasing the pathobionts in the buffalo calf's gut (Varada et al. 2022). Thus, increased lactic acid concentration and lower pH in the gut system create unfavourable conditions that offer resistance to the colonization of potentially infective organisms in the intestinal epithelium of the host, consequently reducing calves’ susceptibility to enteric diseases.

Multiple studies have elucidated the essential role of gut microbiota in the production of SCFAs (lactate, acetate, propionate, and butyrate) by accelerating the fermentation of non-digestible complex dietary fibers in the hindgut of calves (Castro et al. 2016; Song et al. 2018; Oh et al. 2021). Increased calf starter intake may provide a greater luminal substrate for gut microbes to synthesize SCFAs (Jiang et al. 2020). In the present study, we found a significant increase in fecal propionate concentration in the PF-fed group, which indicates better adaptation of host-specific probiotic strains to buffalo calf gut. SCFAs (mainly butyrate) act as a source of energy for the proliferation of enterocytes, colonocytes, and ruminal papillae growth (Varada et al. 2022). Propionate is the major precursor for gluconeogenesis in mammals that accounts for the production of up to 70% of glucose needed to support the daily energy requirements for the basal metabolism of ruminants (Yeoman and White 2014). Song et al. (2018) demonstrated a strong correlation between gut microbiota and SCFA concentration, suggesting a possible interlink between potential probiotic bacteria and the hindgut fermentation profile. In line with our observations, Oh et al. (2021) showed that feeding multispecies probiotics caused a substantial shift in the gut metabolomic signatures, thereby altering SCFA levels in pigs. Ample scientific evidence has recognized that gut microbiome-derived SCFAs may also improve gut barrier integrity by increasing the expression of tight-junction proteins (Nagpal et al. 2018), enhance immune function (Oh et al. 2021), and prevent diarrhea (Na^+^ ions and water absorption) (Binder 2010). Therefore, adequate production of SCFAs by gut microbiota plays a pivotal role in regulating metabolic and physiological homeostasis and favours beneficial microbes' growth, with the ultimate goal of benefiting the gut health of animals (Choudhary et al. 2021).

The GIT microbiome composition of young ruminants and other production animals is considered the key factor underscoring its capability to increase growth performance and, therefore, influence neonatal calves’ health in early life (Amin et al. 2021; Arshad et al. 2021). Early gut microbiota is crucial to the host’s long-term health. The intestinal microbiota of newborn calves changes dynamically during the first several weeks after birth (Malmuthuge et al. 2015). To further explore the effect of probiotic strains on the intestinal flora, we characterized the fecal microbiota of buffalo calves using high-throughput 16S rRNA gene sequencing technology. Adding PF to the calves’ diet caused limited changes within community diversity (α-diversity) from WK0 to WK4, and no statistical differences were observed in the Simpson, Shannon, and Chao1 indices. Our findings are comparable to previous studies on changes in fecal microbiota in calves when fed with multispecies probiotics during the initial eight weeks of life (Wu et al. 2021; Guo et al. 2022). It is well documented that GIT bacterial population go through dynamic changes in abundance and diversity with the progressing age of calves (Amin et al. 2021). The dietary shifts from liquid milk to increased consumption of calf starter may be the reason for age-related shifts in gut bacterial diversity (Zhang et al. 2021). However, bacterial abundance may not represent their accurate function, and that the roles played by bacteria may be more significant than their numbers (Fomenky et al. 2018). Supplementation of probiotics to the calves could modulate the bacterial composition and diversity of the GIT (Cangiano et al. 2020). However, it might have a profound impact on the microbial community composition, but less effect on the diversity (Fomenky et al. 2018; Villot et al. 2019).

Phylum level classification of the 16S rRNA gene amplicon sequencing data revealed that *Bacteroidetes*, *Firmicutes,* and *Proteobacteria* were the same dominant microbial taxa in the developing gut of preweaning calves as compared to the adult animal gut, which was in accordance with the previous reports (Rosa et al. 2021; Guo et al. 2022; Dixit et al. 2022). It has been demonstrated that *Bacteroidetes* are known to play a crucial role in the degradation of starch, fiber, dietary protein, and absorption of amino acids and peptides in the intestine (Malmuthuge et al. 2015). *Firmicutes* are common gut microbes that break down complex carbohydrates that can’t be digested by the host’s enzymes (Choudhary et al. 2021). In this study, the ratio of F/B in the faeces was not statistically different between the two groups. Abnormally increased *Firmicutes* and decreased *Bacteroidetes* abundances are associated with imbalances in the gut microbial ecology (GIT dysbiosis condition) and some dysbiosis-linked diseases, including diarrhea in calves (Fan et al. 2021), obesity, diabetes, and irritable bowel syndrome in humans (Schmidt et al. 2018). Notably, a high relative prevalence of *Firmicutes* is not desirable in the gut due to their negative influence on fat and glucose metabolism (Magne et al. 2020). This finding suggests that administration of host-specific probiotics may ameliorate gut dysbiosis induced diseases by enhancing beneficial *Bacteroidetes* abundance, while suppressing *Firmicutes* count and might serve as potential biotherapy to control/cure diarrhea in neonatal calves. In addition, feeding PF also reduced the abundance of fecal *Proteobacteria*, a major pathogen linked with intestinal diseases (Fan et al. 2021). The phylum *Proteobacteria* contains several opportunistic pathogens such as enteropathogenic *E. coli, Salmonella, Helicobacter pylori,* and *Vibrio cholera* that can cause infectious diarrhea in neonatal calves (Jiang et al. 2020).

At the genus level, perhaps the most interesting result for PF treatment was the significant increase in the population of the *Prevotella* genus compared to the control group. For instance, *Prevotella* can produce propionate, which serves as an important source of energy for the host (Dixit et al. 2022). Thus, higher fecal propionate levels observed in this study may be related to greater abundance of the *Prevotella* genus (Guo et al. 2022). In line with our results, Koringa et al. (2019) detected a maximum abundance of *Prevotella* spp. at around 30 days of buffalo calves’ age, when forage and concentrate were gradually integrated into the diet. A higher prevalence of *Lactobacillus* spp. in fecal samples of newborn calves has been proven to increase ADG, ADMI, FE and lower diarrhea incidence due to antipathogenic activity against diarrhea causing pathogens in preweaned dairy calves (Jiang et al. 2020; Villot et al. 2019; Wu et al. 2021). This finding further supports our hypothesis that adding host-specific probiotics may enhance growth performance by influencing the fecal microbial composition in buffalo calves. Our study is consistent with previous studies that indicate the essential role of these bacteria in maintaining the cattle gut health (Zhang et al. 2021; Dixit et al. 2022). Therefore, it suggests that PF supplementation was able to eliminate numerous pathogens present in the lumen of the hindgut compared to the control group.

The hindgut microbial fermentation plays an important role in providing nutrient and energy to the neonatal calves prior to the complete development of the rumen (Song et al. 2018). However, the rumen microbiome is also a major part of the gut microbial community of ruminants. Previous studies on gut microbiota of pre-weaned calves reported that the composition of the rumen and the fecal microbiomes had been found to be significantly varying with calf age (Amin et al. 2021; Fan et al. 2021; Malmuthuge et al. 2015). Whereas Malmuthuge et al. (2014) reported that the bacterial composition of the cecum and colon was similar to that of the rumen which differed only in their relative abundance in the GIT of 3-week-old pre-weaned calves. Yet, data from rumen samples would represent the foregut microbiome and the data from fecal samples would exhibit mid-gut and hindgut microbiomes (Koringa et al. 2019). The observed bacterial composition in the present study suggests that fecal samples do not adequately represent the complexity of the calf GIT (Malmuthuge et al. 2014). It is therefore essential to focus on both the rumen and the fecal microbiome in order to effectively assess the dynamics of the ruminant gut microbiome and its potential relationships with the host. The rumen samples’ data were not measured in this trial. Hence, further studies should be performed to better understand the impact of potential probiotics on the composition of the foregut and hindgut microbiomes and their correlation on a large number of targeted animals to resolve the limitations of this study.

Based on the present study findings, it may be concluded that administering a host-specific multispecies probiotic to buffalo calves during the first 30 days after birth significantly improved growth performance, morphometric measurements, and reduced the faecal score. The relative abundance of beneficial microflora (*Lactobacillus* and *Prevotella*) in buffalo calves feces tended to increase, and opportunistic pathobionts *Proteobacteria* decreased. Importantly, probiotic intervention has a profound influence on gut health during early-life stages of calves, which is illustrated by the establishment of homogenous, rich, and stable gut microbiome composition using 16S rRNA gene amplicon sequencing approach.

## Supplementary Information

**Additional file 1:** **Figure S1.** Compatibility tests between Ligilactobacillus salivarius BF-17 and Limosilactobacillus reuteri BF-E7 by A) Cross-streak assay and B) Co-culture techniques on the MRS agar plates. **Figure S2.** Rarefaction plot for 24 samples of V3-V4 region at depth of 38000. The image has been plotted against number of sequences per sample in x-axis vs diversity index in y-axis. The samples have been colored by their respective names. **Figure S3.** Microbial diversity indices for the bacterial communities in the feces samples of Murrah buffalo calves supplemented with probiotics as compared to control group. Basal diet with no supplementation (CON), supplemented with L. reuteri BF-E7 + L. salivarius BF-17 (PF; 1g/calf/d). **Table S1.** The relative abundance of bacteria at phylum level in the fecal samples of Murrah buffalo calves supplemented with probiotic formulation (PF) or not (CON) **Table S2.** The relative abundance of bacteria at genus level (average relative abundance>0.1% in at least one group) in the fecal samples of Murrah buffalo calves supplemented with probiotic formulation (PF) or not (CON).

## Data Availability

All data generated or analysed during this study are included in this published article [and its supplementary information files]. The two probiotics strains (*Limosilactobacillus reuteri* BF-E7, Assigned No- MTCC 25412 and *Ligilactobacillus salivarius* BF-17, Assigned No- MTCC 25413) have been deposited to Microbial Type Culture Collection and Gene Bank (MTCC, Chandigarh, India) and MTCC is registered with the World Data Centre for Microorganisms (WDCM). The raw reads were deposited into the NCBI Sequence Read Archive (SRA) database (BioProject Number: PRJNA835612).

## Abbreviations

BW: Body weight
GIT: Gastrointestinal tract
NCD: Neonatal calf diarrhea
CFU: Colony forming unit
CON: Control group
PF: Probiotics-fed group
DMI: Dry matter intake
ADG: Average daily gain
FE: Feed efficiency
NMDS: Non-metric multi-dimensional scaling
SCFA: Short-chain fatty acids
OUT: Operational Taxonomic Unit
LAB: Lactic acid bacteria
F/B: The ratio of *Firmicutes*/*Bacteroidetes*
