# Supplementary material for: Host-specific probiotics feeding influence growth, gut microbiota, and fecal biomarkers in buffalo calves
Source: AMB Express. 2022 Sep 14;12:118. doi: 10.1186/s13568-022-01460-4 (PMC9475018; doi:10.1186/s13568-022-01460-4)
Supplement: Supplementary file 1 — Additional file 1: Figure S1. Compatibility tests between Ligilactobacillus salivarius BF-17 and Limosilactobacillus reuteri BF-E7 by A) Cross-streak assay and B) Co-culture techniques on the MRS agar plates. Figure S2. Rarefaction plot for 24 samples of V3-V4 region at depth of 38000. The image has been plotted against number of sequences per sample in x-axis vs diversity index in y-axis. The samples have been colored by their respective names. Figure S3. Microbial diversity indices for the bacterial communities in the feces samples of Murrah buffalo calves supplemented with probiotics as compared to control group. Basal diet with no supplementation (CON), supplemented with L. reuteri BF-E7 + L. salivarius BF-17 (PF; 1g/calf/d). Table S1. The relative abundance of bacteria at phylum level in the fecal samples of Murrah buffalo calves supplemented with probiotic formulation (PF) or not (CON) Table S2. The relative abundance of bacteria at genus level (average relative abundance>0.1% in at least one group) in the fecal samples of Murrah buffalo calves supplemented with probiotic formulation (PF) or not (CON). [file 13568_2022_1460_MOESM1_ESM.pdf]

AMB Express

Supplementary Information (SI)

Host-specific probiotics feeding influence growth, gut microbiota, and fecal biomarkers in buffalo calves

Vinay Venkatesh Varada[1] · Sachin Kumar[1*] · Supriya Chhotaray[2] · Amrish Kumar Tyagi[1, 3]

[1]Rumen Biotechnology Lab., Animal Nutrition Division, ICAR-National Dairy Research Institute, Karnal-132001, Haryana, INDIA

[2]Buffalo Breeding Lab, Animal Genetics and Breeding Division, ICAR-National Dairy Research Institute, Karnal-132001, Haryana, INDIA

[3]Present Address: Assistant Director General (Animal Nutrition and Physiology), Indian Council of Agricultural Research, New Delhi, INDIA

***Corresponding author:** email: arensachin@gmail.com (Sachin Kumar); Phone number: +91-1842259069

The PDF file includes:

Figure S1 Compatibility tests between *Ligilactobacillus salivarius* BF-17 and *Limosilactobacillus reuteri* BF-E7 by A) Cross-streak assay and B) Co-culture techniques on the MRS agar plates

Figure S2 Rarefaction plot for 24 samples of V3-V4 region at depth of 38000. The image has been plotted against number of sequences per sample in x-axis vs diversity index in y-axis. The samples have been coloured by their respective names

Figure S3 Microbial diversity indices for the bacterial communities in the feces samples of Murrah buffalo calves supplemented with probiotics as compared to control group. Basal diet with no supplementation (CON), supplemented with *L. reuteri* BF-E7 + *L. salivarius* BF-17 (PF; 1g/calf/d)

Table S1 The relative abundance of bacteria at phylum level in the fecal samples of Murrah buffalo calves supplemented with probiotic formulation (PF) or not (CON)

Table S2 The relative abundance of bacteria at genus level (average relative abundance>0.1% in at least one group) in the fecal samples of Murrah buffalo calves supplemented with probiotic formulation (PF) or not (CON)

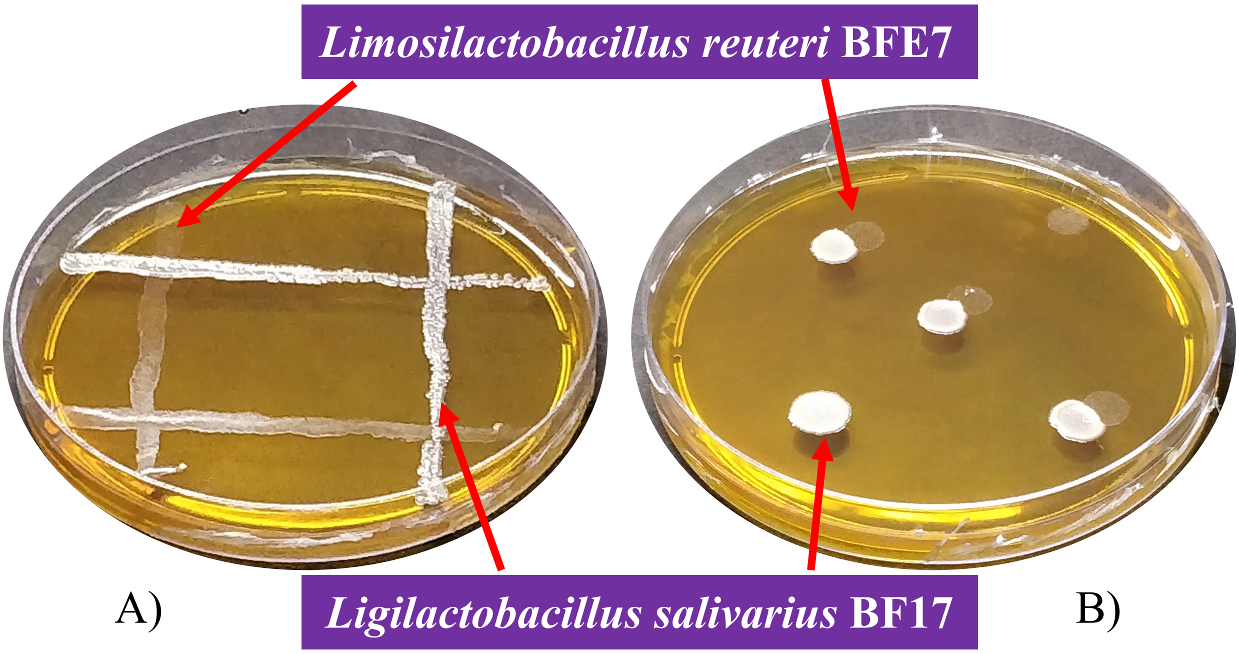

Figure S1 Compatibility tests between *Ligilactobacillus salivarius* BF-17 and *Limosilactobacillus reuteri* BF-E7 by **A)** Cross-streak assay and **B)** Co-culture techniques on the MRS agar plates

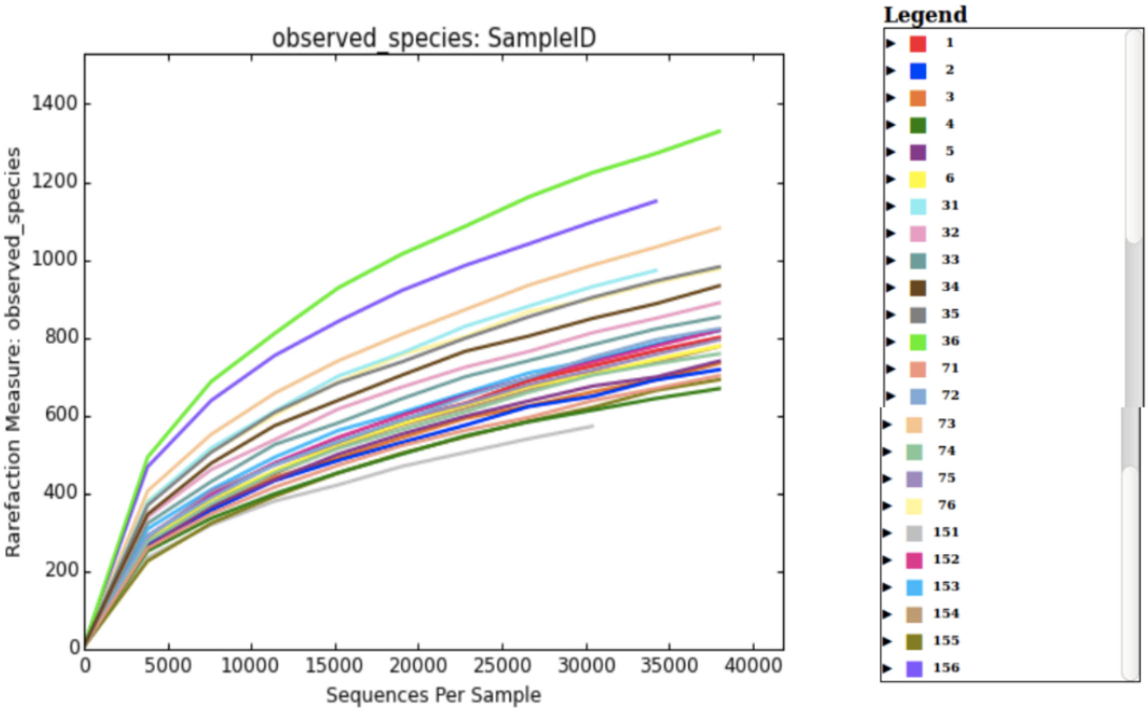

Figure S2 Rarefaction plot for 24 fecal samples of V3-V4 region at depth of 38000. The image has been plotted against number of sequences per sample in x-axis vs diversity index in y-axis. The samples have been coloured by their respective names

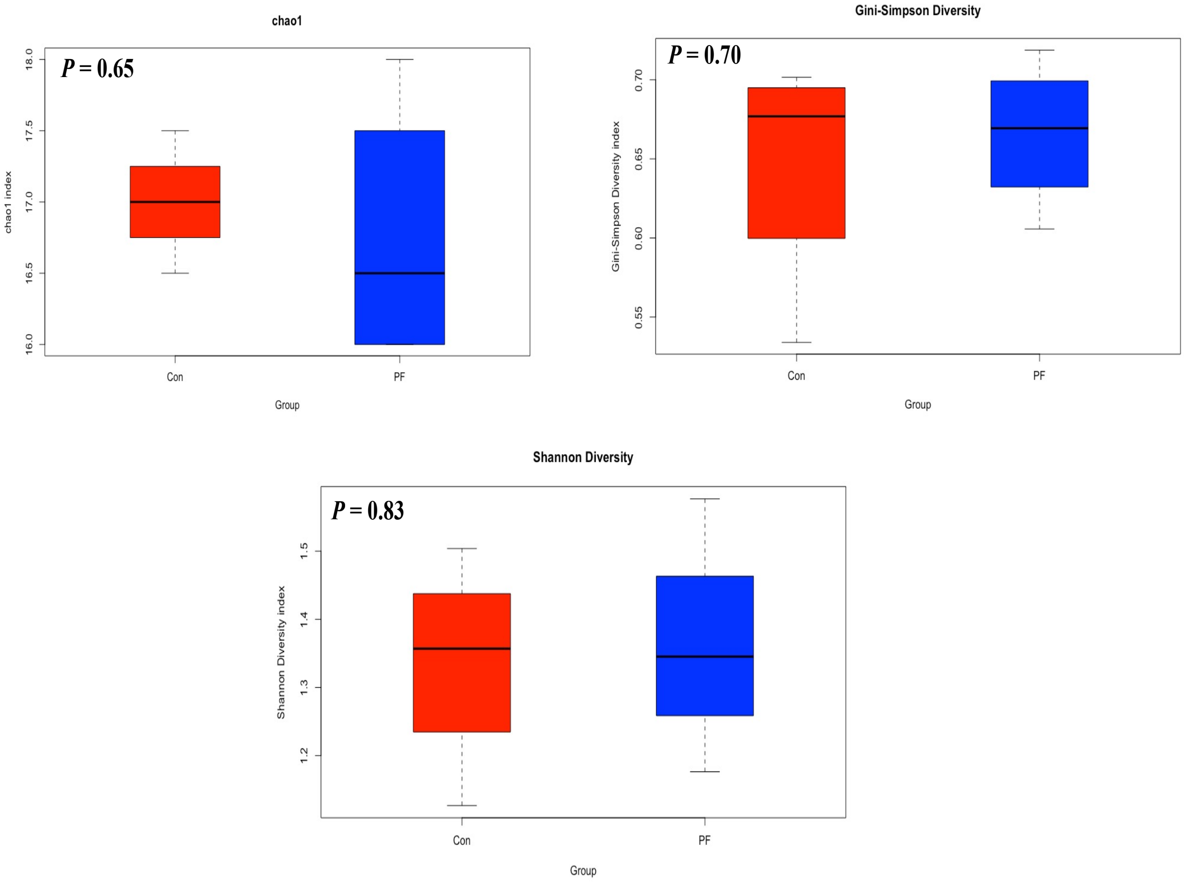

Figure S3 Microbial diversity indices for the bacterial communities in the feces samples of Murrah buffalo calves supplemented with probiotics as compared to control group. Basal diet with no supplementation (CON), supplemented with *L. reuteri* BF-E7 + *L. salivarius* BF-17 (PF; 1g/calf/d)

Table S1 The relative abundance of bacteria at phylum level in the fecal samples of Murrah buffalo calves supplemented with probiotic formulation (PF) or not (CON)

Phylum	Treatment (Trt)[1]		P Value (Trt)
	CON	PF	
Bacteroidetes			
Day 0	65.45±13.65	63.14±9.96	0.43
Day 7	40.45±12.55	47.81±1.07	0.21
Day 15	45.18±13.90	46.95±9.25	0.38
Day 30	59.30±7.05	61.23±21	0.44
Average	52.59±11.73	54.78±8.59	0.18
Firmicutes			
Day 0	27.14±13.30	30.20±11.47	0.41
Day 7	46.33±0.92	51.98±12.97	0.27
Day 15	43.22±12.22	44.86±13.63	0.35
Day 30	30.32±1.59	30.70±13.24	0.48
Average	33.29±15.02	39.44±10.78	0.10
Actinobacteria			
Day 0	1.48±1.85	0.65±0.41	0.23
Day 7	0.77±0.49	1.37±0.48	0.17
Day 15	0.24±0.27	0.38±0.18	0.08
Day 30	0.15±0.09	0.14±0.13	0.47
Average	0.66±0.61	0.64±0.54	0.46
Proteobacteria			
Day 0	3.92±1.66	3.91±2.74	0.50
Day 7	3.53±0.52	4.62±1.45	0.09
Day 15	7.45^{b}±2.38	2.75^{a}±0.28	0.03
Day 30	2.23±0.26	2.63±1.90	0.39
Average	4.28±2.23	3.48±0.96	0.29
Cyanobacteria			
Day 0	1.13±0.72	1.87±1.32	0.30
Day 7	0.97±1.11	0.15±0.17	0.18
Day 15	6.15^{a}±3.03	1.61^{b}±0.98	0.04
Day 30	6.33±5.40	4.58±7.53	0.40
Average	3.65±3.00	2.05±1.85	0.12
Fusobacteria			
Day 0	0.83±0.92	0.18±0.17	0.20
Day 7	0.37±0.62	1.21±1	0.13
Day 15	0.18±0.19	0.27±0.41	0.30
Day 30	1.26±2.18	0.24±0.20	0.27
Average	0.66±0.48	0.47±0.49	0.34

[1]Treatments: [†]Basal diet with no supplementation (CON), supplemented with *L. reuteri* BF-E7 + *L. salivarius* BF-17 (PF; 1g/calf/d); [ab]Means bearing different letters in a row differ significantly (*P* <0.05); Trt, treatment; Values are expressesd as mean±SD; P<0.05 was regarded as statistically significant, and 0.05<P<0.10 was regarded as a statistical tendency

Table S2 The relative abundance of bacteria at genus level (average relative abundance>0.1% in at least one group) in the fecal samples of Murrah buffalo calves supplemented with probiotic formulation (PF) or not (CON)

Genus	Treatment (Trt)[1]		P Value (Trt)
	CON	PF	
Anaerovibrio			
Day 0	0.57±0.08	0.46±0.42	0.32
Day 7	1.65±1.53	0.79±0.43	0.25
Day 15	0.46±0.41	0.54±0.93	0.46
Day 30	0	0.52±0.55	0.12
Average	0.67±0.70	0.58±0.15	0.38
Bacteroides			
Day 0	15.65±8.48	15.13±8.38	0.47
Day 7	1.55±1.56	7.80±10.29	0.17
Day 15	11.50±12.32	14.68±6.10	0.40
Day 30	7.60±5.04	18.63±22.43	0.28
Average	9.08±6.00	14.06±4.53	0.07
Coprococcus			
Day 0	1.22±1.20	2.07±1.02	0.30
Day 7	0.90±1.18	0.28±0.48	0.26
Day 15	0.62±0.23	0.00	0.02
Day 30	0.32±0.56	0.24±0.42	0.21
Average	0.77±0.38	0.65±0.96	0.38
Faecalibacterium			
Day 0	9.02±7.70	9.97±9.12	0.46
Day 7	6.18±4.02	4.92±1.08	0.34
Day 15	7.33±4.35	8.83±5.05	0.40
Day 30	6.10±2.71	6.07±4.35	0.50
Average	7.16±1.36	7.45±2.35	0.33
Lactobacillus			
Day 0	0.37±0.38	1.31±1.06	0.08
Day 7	5.00±3.65	8.06±11.26	0.30
Day 15	1.44±0.47	5.00±5.56	0.18
Day 30	1.59±2.22	0.95±0.66	0.33
Average	2.1±2.01	3.88±3.39	0.08
Oscillospira			

Day 0	2.47±2.25	5.83±8.8.25	0.31
Day 7	2.87±0.91	2.44±0.84	0.07
Day 15	3.15[a]±1.18	1.17[b]±0.06	0.05
Day 30	1.18±1.22	2.69±0.76	0.11
Average	2.42±0.87	3.03±1.98	0.31
Prevotella			
Day 0	20.72±17.96	29.23±11.05	0.32
Day 7	13.40±2.12	16.48±10.33	0.31
Day 15	3.39±4.28	7.18±7.84	0.29
Day 30	18.25±14.89	26.51±25.55	0.37
Average	13.94[a]±7.66	19.85[b]±10.07	0.01
Ruminococcus			
Day 0	0.62±0.71	0.21±0.37	0.25
Day 7	0.20±0.35	0.47±0.42	0.30
Day 15	0.81±0.74	1.11±0.47	0.32
Day 30	0.21±0.36	0.26±0.23	0.40
Average	0.46±0.30	0.51±0.41	0.38
Sutterella			
Day 0	1.50±0.85	2.38±1.47	0.23
Day 7	5.37±3.73	2.21±0.38	0.12
Day 15	2.75±0.97	2.31±0.03	0.25
Day 30	1.90±2.09	1.00±0.42	0.24
Average	2.88±1.74	1.98±0.65	0.18

[1]Treatments: [†]Basal diet with no supplementation (CON), supplemented with *L. reuteri* BF-E7 + *L. salivarius* BF-17 (PF; 1g/calf/d); [ab]Means bearing different letters in a row differ significantly (*P* <0.05); Trt, treatment; Values are expressed as mean±SD; P<0.05 was regarded as statistically significant, and 0.05<P<0.10 was regarded as a statistical tendency
